# Enhanced glutamine uptake influences composition of immune cell infiltrates in breast cancer

**DOI:** 10.1038/s41416-019-0626-z

**Published:** 2019-12-10

**Authors:** Rokaya El Ansari, Madeleine L. Craze, Maryam Althobiti, Lutfi Alfarsi, Ian O. Ellis, Emad A. Rakha, Andrew R. Green

**Affiliations:** 1 Nottingham Breast Cancer Research Centre, Division of Cancer and Stem Cells, School of Medicine, University of Nottingham, Nottingham City Hospital, Hucknall Road, Nottingham, NG5 1PB UK; 2 Department of Pathology, Faculty of Medicine, University of Tripoli, Tripoli, Libya; 3 Department of Clinical Laboratory Science, College of Applied Medical Science, Shaqra University 33, Shaqra, 11961 Saudi Arabia

**Keywords:** Breast cancer, Proteomics

## Abstract

**Background:**

Cancer cells must alter their metabolism to support proliferation. Immune evasion also plays a role in supporting tumour progression. This study aimed to find whether enhanced glutamine uptake in breast cancer (BC) can derive the existence of specific immune cell subtypes, including the subsequent impact on patient outcome.

**Methods:**

SLC1A5, SLC7A5, SLC3A2 and immune cell markers CD3, CD8, FOXP3, CD20 and CD68, in addition to PD1 and PDL1, were assessed by using immunohistochemistry on TMAs constructed from a large BC cohort (*n* = 803). Patients were stratified based on SLC protein expression into accredited clusters and correlated with immune cell infiltrates and patient outcome. The effect of transient siRNA knockdown of SLC7A5 and SLC1A5 on PDL1 expression was evaluated in MDA-MB-231 cells.

**Results:**

High SLCs were significantly associated with PDL1 and PD1 +, FOXP3 +, CD68 + and CD20 + cells (*p* < 0.001). Triple negative (TN), HER2 + and luminal B tumours showed variable associations between SLCs and immune cell types (*p* ≤ 0.04). The expression of SLCs and PDL1, PD1 +, FOXP3 + and CD68 + cells was associated with poor patient outcome (*p* < 0.001). Knockdown of SLC7A5 significantly reduced PDL1 expression.

**Conclusion:**

This study provides data that altered glutamine pathways in BC that appears to play a role in deriving specific subtypes of immune cell infiltrates, which either support or counteract its progression.

## Background

Altered metabolic pathways are readily accepted as part of the revised hallmarks of cancer where cancer cells adapt their metabolism in order to resist the unfavourable, nutrient-deprived conditions and to respond to the increased energy demands required by their unremitting proliferation.^1^ Many cancer cells are highly reliant on amino acids for their growth, not only because they are precursors for nucleotide and protein synthesis, but also because they activate mammalian target of rapamycin complex1 (mTORC1) through nutrient-signalling pathways, which in turn regulates protein translation and cell growth.^2,3^

Solute carrier family 1 member 5 (SLC1A5) and solute carrier family 7 member 5 (SLC7A5) are two key amino acid transporters that have been attracting attention due to their role in supporting tumour metabolism. Primarily, SLC1A5 maintains the sodium‐coupled influx of glutamine, whereas SLC7A5 mediates the efflux of this amino acid in exchange with the influx of leucine, an essential amino acid and potent activator of mTORC1.^4,5^ SLC7A5 requires a covalent association with the heavy chain of SLC3A2, for its functional expression in plasma membrane.^6^ We have previously described the potential utility of these transporters as prognostic factors in certain BC subtypes.^7,8^ Further, we have recently stratified BC patients into three accredited clusters based on the protein expression of these three solute carriers.^9^

The role of the tumour microenvironment (TME) is well known with respect to disease development and progression. One of the important components of the TME is immune cells, including the regulatory T cells (Treg) and tumour-associated macrophages (TAM), which gain pro-tumoural functions stimulating tumour growth, progression, invasion and metastasis. Conversely, other immune cells such as CD8 + and CD20 + lymphocytes are responsible for anti-tumoural responses by activating host defence mechanisms preventing immune evasion.^10,11^ Immune evasion is a strategy used by tumours to evade a host’s immune response in an attempt to maximise their probability to continue surviving and growing. Tumour immune evasion includes several mechanisms such as progressive formation of an immune-suppressive environment within the tumour and the selection of tumour variants resistant to immune effectors (immunoediting).^12^

Composition of the inflammatory cell infiltrates in BC also correlates with clinical outcome, where an abundant infiltration of pro-tumorigenic cells is associated with poor outcome, while TME enriched in cells with anti-tumorigenic functions has a favourable effect on patient survival.^13–15^ Programmed cell death 1 (PD1) and its activating ligand, programmed death ligand 1 (PDL1) act in attenuating the anticancer immune response and promoting T-regulatory cell development and function. Indeed, PDL1 is expressed by tumour cells of several cancer types, with evidence of an association with aggressive tumour behaviour and poor prognosis.^16–20^

Previous studies show that the cellular contents of the TME change in parallel with tumour growth/progression, and the accompanied alterations in glucose metabolism in cancer cells are tightly linked to the composition of the surrounding immune cells.^21,22^ We therefore hypothesise that the reprogramming of glutamine metabolism will have an impact on the structure of the immune cells. This study aimed to determine whether overexpression of the key glutamine solute carriers can derive the existence of specific subtypes of immune cells, in addition to their supportive role in estimating the clinical outcome.

## Methods

### Patient cohort

This study evaluated a well-characterised cohort of early-stage, primary operable, invasive BC patients aged ≤70 years. Patients (*n* = 803) presented at Nottingham City Hospital from 1989 to 1998. Patient management was uniform and based on tumour characteristics by Nottingham Prognostic Index (NPI) and hormone receptor status. Clinical history, tumour characteristics, information on therapy and outcomes are prospectively maintained. Outcome data included development and time to distant metastasis (DM) and breast cancer-specific survival (BCSS), defined as the time (in months) from the date of primary surgical treatment to the time of death from BC. The clinicopathological parameters for the BC series are summarised in Supplementary Table 1.

### Tissue microarrays (TMAs) and immunohistochemistry

TMAs consisting of 0.6-mm tumour tissue cores were arrayed and immunohistochemically profiled for SLC1A5, SLC7A5, SLC3A2, PD1, PDL1, CD3, CD8, CD68, CD20 and FOXP3, as previously described.^23,9,13,24^

TMA sections stained with SLC1A5, SLC7A5, SLC3A2, PDL1 and PD1 were scanned by using high-resolution digital images (NanoZoomer; Hamamatsu Photonics, Welwyn Garden City, UK), at ×20 magnification. Evaluation of staining was based on a semi-quantitative assessment by using a modified histochemical score (H-score)^25^ as previously described.^9^ Clustering analysis of SLC1A5, SLC7A5 and SLC3A2 protein expression was previously performed by using two algorithms, partitioning around medoids (PAM) and K-means, to stratify tumours into the optimal number of clusters based on their H-score. The three clusters were characterised as follows: low SLCs (SLC1A5–/SLC7A5–/SLC3A2–), high SLC1A5 (SLC1A5 +/SLC7A5–/SLC3A2–) and high SLCs (SLC1A5 +/SLC7A5 +/SLC3A2 + ).^9^

Immunohistochemical detection of a panel of lymphocyte markers including pan-T-cell CD3, cytotoxic T-cell CD8, T-reg FOXP3, B-cell CD20 and histiocytic cell marker CD68 was previously determined, and the total number of each immune cell type was counted in each tumour core by using a Nikon Eclipse 80i microscope (Nikon, Tokyo, Japan) as described.^13–15^ BC molecular subtypes were defined, based on tumour IHC profile and the Elston–Ellis^26^ mitotic score as ER +/HER2– low proliferation (mitotic score 1), ER +/HER2– high proliferation (mitotic scores 2 and 3) and HER2-positive class: HER2 + regardless of ER status, triple negative (TN): ER–, PgR– and HER2–.^27^

### siRNA transfection of SLC7A5 and SLC1A5

The TN cell line, MDA-MB-231, was obtained from American Type Culture Collection; Rockville, MD, USA and cultured in Roswell Park Memorial Institute (RPMI-1640) medium (Sigma-Aldrich, UK) supplemented with 10% foetal bovine serum (Sigma-Aldrich, UK). Mycoplasma testing was carried out on a routine basis by using the MycoAlert Detection kit (R&D Systems). In total, 5 × 10^4^ cells were seeded per well in a 24-well plate and transfected by using the reverse transfection method with 25 and 100 pmol siRNA (ThermoFisher Scientific), for SLC7A5 and SLC1A5, respectively, and lipofectamine (RNAiMAX) according to the manufacturer’s protocol.

siRNA antisense sequences were as follows: 5′-UUGGGAUCUAGAUUGGACAca-3′ (for SLC7A5) and 5′-AAAGAGUAAACCCACAUCCtc-3′ (for SLC1A5). Untransfected cells were carried out alongside the experiment as controls. SLC7A5 and SLC1A5 expression of transfected cells was performed in duplicate and determined by Western blotting analysis (Supplementary Fig. 2A–B).

### Statistical analysis

Statistical analysis was performed by using SPSS 24.0 statistical software (SPSS Inc., Chicago, IL, USA). The chi-square test was performed for interrelationships between categorical variables. Differences between two groups of normalised data were assessed by using t test. Survival curves were analysed by Kaplan–Meier with log-rank test. This was performed with BC- specific death; those who died of other causes, alive and lost to follow-up were censored. *P*-values were adjusted by using Bonferroni correction for multiple testing. A *p*-value  < 0.05 was considered significant. The study endpoints were 10-year breast cancer-specific survival (BCSS) or distant metastasis-free survival (DMFS).

This study was approved by the Nottingham Research Ethics Committee 2 under the title ‘Development of a molecular genetic classification of breast cancer’ and the North West– Greater Manchester Central Research Ethics Committee under the title ‘Nottingham Health Science Biobank (NHSB)’ reference number 15/NW/0685.

## Results

### Expression of SLC1A5, SLC7A5, SLC3A2 and immune cell markers in BC

**Fig. 1:**
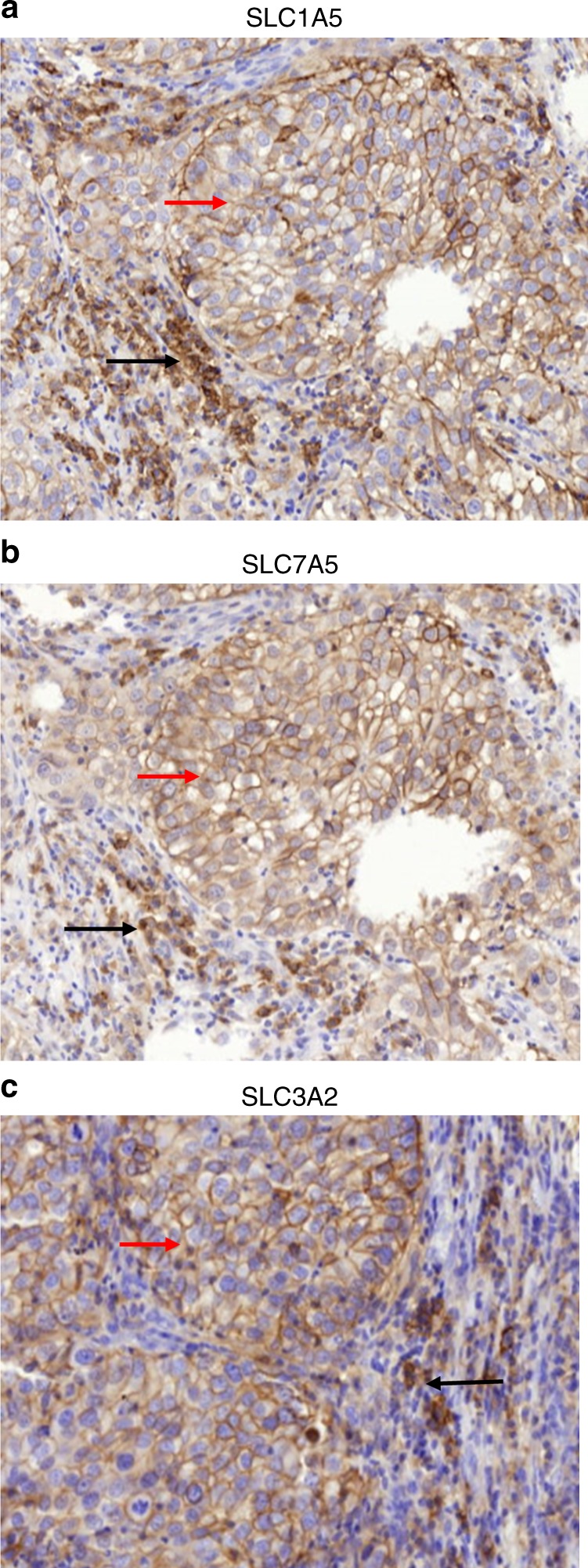
The expression of solute carriers **a** SLC1A5, **b** SLC7A5 and **c** SLC3A2 was predominant in the cell membrane of the breast cancer cells (red arrows) and the adjacent immune cell infiltrates (black arrows)

Expression of the three solute carriers was predominantly in the membrane of the invasive BC cells, with intensity levels varying from absent to high. High expression of the solute carriers was also observed in lymphocytic infiltrates, which were in the stroma adjacent to the tumour cells (Fig. 1a–c). Immunohistochemical expression of solute carriers and immune cell markers in invasive BC cores is illustrated in Supplementary Fig. 1.

### Association of SLCs with immune cell infiltrates

Tumour-infiltrating FOXP3 + lymphocytes and CD68 + macrophages were both predominant in tumours with high SLCs and to a lesser extent in those tumours with high SLC1A5 expression (Table 1, *p* < 0.0001). CD20 + lymphocytes were mainly observed in tumours with high SLC expression (Table 1, *p* < 0.0001). While PDL1 was highly expressed in tumours with high SLCs and high SLC1A5 expression, PD1 + cells were mainly expressed with tumours with high SLC expression (Table 1, *p* < 0.0001). In contrast, there was no significant association between the SLC clusters and CD3 + or CD8 + T lymphocytes (Table 1, *p* = 0.84 and *p* = 0.24), respectively.

**Table 1:** Association of SLCs with different subtypes of immune cell markers

Immune cell marker	Low SLCs, *n* (%)	High SLC1A5, *n* (%)	High SLCs, *n* (%)	χ^2^ (*p*-value)	Adjusted *p*-value
*CD3*					
Negative	36 (56.3)	21 (32.8)	7 (10.9)	1.7	
Positive	268 (54.1)	142 (28.7)	85 (17.2)	(0.42)	0.84
*CD8*					
Negative	71 (57.7)	35 (28.5)	17 (13.8)	4.9	
Positive	236 (48.9)	140 (29.0)	107 (22.2)	(0.08)	0.24
*FOXP3*					
Negative	148 (63.8)	67 (28.9)	17 (7.3)	37.3	
Positive	188 (44.9)	122 (29.1)	109 (26.0)	(7.9 × 10^–9^)	**<0.0001**
*CD68*					
Negative	139 (67.1)	47 (22.7)	21 (10.1)	35	
Positive	165 (42.7)	124 (32.1)	97 (25.1)	(2.5 × 10^–8^)	**<0.0001**
*CD20*					
Negative	255 (55.4)	134 (29.1)	71 (15.4)	17.1	
Positive	81 (44.5)	47 (25.8)	54 (29.7)	(0.0001)	**0.0004**
*PD1*					
Negative	203 (52.1)	111 (28.5)	76 (19.5)	42.5	
Positive	134 (36.1)	85 (22.9)	152 (41.0)	(6.0 × 10^–10^)	**<0.0001**
*PDL1*					
Negative	81 (67.5)	28 (23.3)	11 (9.2)	16.8	
Positive	287 (48.2)	178 (29.9)	130 (21.8)	(0.0002)	**0.0004**

Bold values indicate statistical significance

### Association between SLC clusters and immune cell infiltrates varied among BC molecular subtypes

CD68 + cells were significantly associated with high SLC expression in both ER + high proliferative/luminal B and TN subtypes (Tables 2, 3, *p* = 0.02 and *p* = 0.03), respectively. In contrast, FOXP3 + cells were only associated within TN tumours showing high SLC expression (Table 3, *p* = 0.02). CD20 + cells were only observed in ER + high proliferative/luminal B tumours with high SLCs (Table 2, *p* = 0.04). PD1 and PDL1 were also significantly expressed in HER2 + tumours with high SLC expression (Table 3, *p* = 0.03 and *p* = 0.04), respectively. However, within TN tumours, only PDL1 expression was associated with high SLC expression (Table 3, *p* = 0.04). There were no significant associations between the SLC clusters and immune cell infiltrates in ER + low proliferative/luminal A tumours (Table 2).

**Table 2:** Association of SLCs with immune cell markers in ER + low proliferative/luminal A and ER + high proliferative/luminal B BC subtypes

Immune cell marker	ER + low proliferative/luminal A					ER + high proliferative/luminal B				
	Low SLCs, *n* (%)	High SLC1A5, *n* (%)	High SLCs, *n* (%)	χ^2^ (*p*-value)	Adjusted *p*-value	Low SLCs, *n* (%)	High SLC1A5, *n* (%)	High SLCs, *n* (%)	χ^2^ (*p*-value)	Adjusted *p*-value
*CD3*										
Negative	10 (66.7)	4 (26.7)	1 (6.7)	1.41	3.92	10 (41.7)	12 (50.0)	2 (8.3)	1.31	4.08
Positive	95 (79.8)	20 (16.8)	4 (3.4)	(0.49)		93 (52.5)	67 (37.9)	17 (9.6)	(0.51)	
*CD8*										
Negative	22 (75.9)	5 (17.2)	2 (6.9)	0.22	6.23	20 (47.6)	16 (38.1)	6 (14.3)	0.56	5.25
Positive	82 (78.8)	17 (16.3)	5 (4.8)	(0.89)		84 (50.6)	65 (39.2)	17 (10.2)	(0.75)	
*FOXP3*										
Negative	61 (80.3)	12 (15.8)	3 (3.9)	0.94	3.72	47 (55.3)	34 (40.0)	4 (4.7)	3.76	0.9
Positive	50 (73.5)	14 (20.6)	4 (5.9)	(0.62)		69 (51.1)	49 (36.3)	17 (12.6)	(0.15)	
*CD68*										
Negative	55 (85.9)	7 (10.9)	2 (3.1)	4.75	0.45	50 (66.7)	21 (28.0)	4 (5.3)	10.6	**0.02**
Positive	47 (70.1)	15 (22.4)	5 (7.5)	(0.09)		56 (43.8)	54 (42.2)	18 (14.1)	(0.005)	
*CD20*										
Negative	92 (79.3)	19 (16.4)	5 (4.3)	0.56	3.00	83 (50.3)	69 (41.8)	13 (7.9)	8.03	**0.04**
Positive	20 (74.1)	5 (18.5)	2 (7.4)	(0.75)		32 (60.4)	12 (22.6)	9 (17.0)	(0.01)	
*PD1*										
Negative	85 (79.4)	19 (17.8)	3 (2.8)	3.01	0.66	64 (50.8)	53 (42.1)	9 (7.1)	6.02	0.12
Positive	42 (71.2)	12 (20.3)	5 (8.5)	(0.22)		54 (50.5)	35 (32.7)	18 (16.8)	(0.04)	
*PDL1*										
Negative	24 (82.8)	4 (13.8)	1 (3.4)	0.6	1.46	19 (70.4)	6 (22.2)	2 (7.4)	3.64	0.3
Positive	99 (76.2)	24 (18.5)	7 (5.4)	(0.73)		109 (0.9)	80 (37.4)	25 (11.7)	(0.15)	

Bold values indicate statistical significance

**Table 3:** Association of SLCs with immune cell markers in HER2 + and triple-negative BC subtypes

Immune cell marker	HER2+					Triple negative				
	Low SLCs, *n* (%)	High SLC1A5, *n* (%)	High SLCs, *n* (%)	χ^2^ (*p*-value)	Adjusted *p*-value	Low SLCs, *n* (%)	High SLC1A5, *n* (%)	High SLCs, *n* (%)	χ^2^ (*p*-value)	Adjusted *p*-value
*CD3*										
Negative	3 (75.0)	1 (25.0)	0 (0.0)	3.02	1.76	3 (30.0)	4 (40.0)	3 (30.0)	4.68	0.72
Positive	22 (33.8)	26 (40.0)	17 (26.2)	(0.22)		16 (25.4)	9 (14.3)	38 (60.3)	(0.09)	
*CD8*										
Negative	5 (62.5)	3 (37.5)	0 (0.0)	4.37	0.77	6 (30.0)	7 (35.0)	7 (35.0)	7.72	0.14
Positive	23 (31.1)	30 (40.5)	21 (28.4)	(0.11)		18 (21.2)	11 (12.9)	56 (65.9)	(0.02)	
*FOXP3*										
Negative	6 (46.2)	6 (46.2)	1 (7.7)	2.47	1.74	9 (42.9)	6 (28.6)	6 (28.6)	9.86	**0.02**
Positive	22 (30.6)	31 (43.1)	19 (26.4)	(0.29)		18 (19.8)	13 (14.3)	60 (65.9)	(0.007)	
*CD68*										
Negative	4 (26.7)	4 (26.7)	7 (46.7)	5.18	0.35	3 (20.0)	7 (46.7)	5 (33.3)	10.1	**0.03**
Positive	24 (35.3)	31 (45.6)	13 (19.1)	(0.07)		18 (21.4)	11 (13.1)	55 (65.5)	(0.007)	
*CD20*										
Negative	20 (42.6)	17 (36.2)	10 (21.3)	3.05	0.84	13 (21.7)	13 (21.7)	34 (56.7)	1.55	1.84
Positive	9 (24.3)	18 (48.6)	10 (27.0)	(0.21)		12 (25.0)	6 (12.5)	30 (62.5)	(0.46)	
*PD1*										
Negative	14 (37.8)	20 (54.1)	3 (8.1)	8.57	0.03	11 (29.7)	7 (18.9)	19 (51.4)	1.35	1.5
Positive	16 (29.1)	20 (36.4)	19 (34.5)	(0.01)		19 (20.4)	18 (19.4)	56 (60.2)	(0.50)	
*PDL1*										
Negative	8 (47.1)	9 (52.9)	0 (0.0)	7.4	0.04	7 (50.0)	3 (21.4)	4 (28.6)	7.48	0.04
Positive	19 (26.0)	32 (43.8)	22 (30.1)	(0.02)		21 (19.4)	20 (18.5)	67 (62.0)	(0.02)	

Bold values indicate statistical significance

### The co-occurrence of SLCs and immune cell infiltrates correlates with patient outcome

**Fig. 2:**
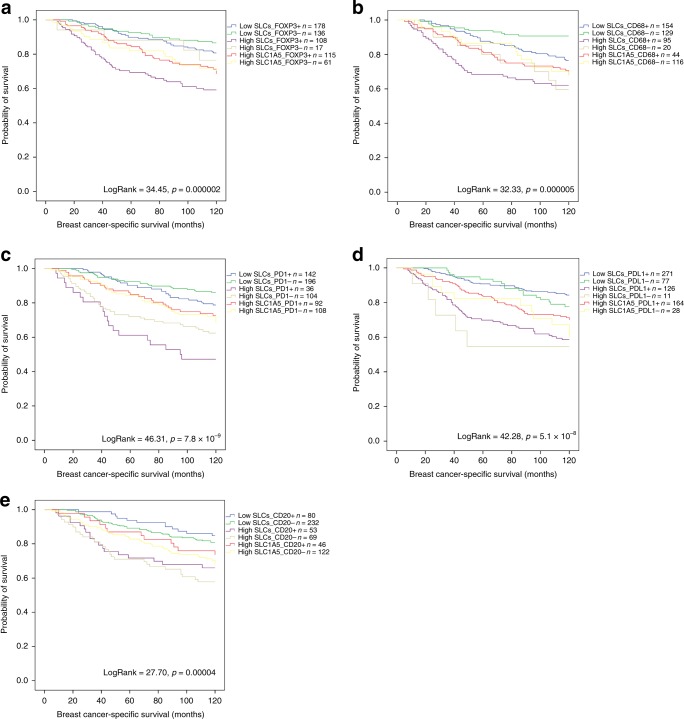
Breast cancer-specific survival in SLCs and immune marker co-expression. **a** SLCs and FOXP3. **b** SLCs and CD68. **c** SLCs and PD1. **d** SLCs and PDL1. **e** SLCs and CD20

Variable associations with patient outcome were observed when investigating the co-occurrence of the SLC clusters with the immune cell markers. The coexistence of high SLCs with FOXP3 + T lymphocytes was predictive of a shorter BCSS (Fig. 2a, *p* < 0.001). Similar associations were observed when CD68 + macrophages, PD1 + cells and PDL1 expression were considered (Fig. 2b–d, *p* < 0.001). However, patients with tumours showing both high SLCs and CD20 + lymphocytes, showed better BCSS (Fig. 2e, *p* < 0.001).

There was a comparable observation regarding the association of SLCs and immune cell markers with DMFS, where high SLC expression accompanied by the presence of FOXP3 +, CD68 + and PD1 + cells or PDL1 expression showed significantly shorter DMFS (Supplementary Fig. 3A–D, all *p* < 0.001). In contrast, the presence of CD20 + lymphocytes and high SLCs conferred a longer DMFS (Supplementary Fig. 3E, *p* = 0.002).

### SLC7A5 plays a role in PDL1 expression in TNBC

**Fig. 3:**
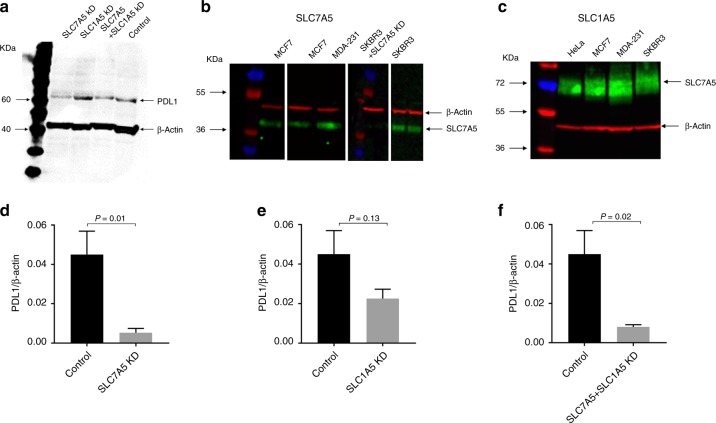
PDL1 protein expression in western blotting. **a** Western blot analysis of PDL1 protein expression in MDA-MB-231 cells transfected with SLC7A5 and/or SLC1A5 SiRNA. Western blot results in different BC cell lysates for **b** SLC7A5 and **c** SLC1A5. The bar graph summarises the expression levels of PDL1 protein, by using β-actin as normalised control, upon **d** SLC7A5 SiRNA transfection. **e** SLC1A5 SiRNA transfection. **f** SLC7A5 and SLC1A5 SiRNA transfection. Data represent the mean and error bars of three independent experiments

Functional analysis was carried out by using the TNBC cell line, MDA-MB-231, due to the high expression of PDL1 together with SLC7A5 and/or SLC1A5 expression, and also based on the significant associations found between SLCs and immune cell markers, including PDL1, within the TNBC subtype (Fig. 3b, c).

siRNA knockdown of SLC1A5 or SLC7A5 in MDA-MB-231 reduced the protein expression of PDL1. However, this observation was only significant upon targeting SLC7A5 (Fig. 3d, *p* = 0.01) but not SLC1A5 (Fig. 3e, *p* = 0.13). Significant reduction of PDL1 protein expression was also observed in cells transfected with both SLC1A5 and SLC7A5 siRNAs (Fig. 3f, *p* = 0.02).

## Discussion

Breast cancer is a heterogeneous disease with various subtypes^28^ that are different in terms of morphology, molecular profiles, response to therapy and clinical behaviour. Breast cancer also shows heterogeneity in metabolic reprogramming, where highly proliferative tumours are distinguished based on their metabolic signatures.^29–31^

Cancer cells undergo metabolic changes in order to satisfy the demands of necessary energy and cellular building blocks. One of the most prominent is the increase in glutamine consumption, which is reflected by the upregulation of the key glutamine transporters (SLC1A5 and the SLC7A5–SLC3A2 dimeric complex) at the surface of the tumour cells. We have recently demonstrated that the combined expression of the three solute carriers (SLC1A5, SLC7A5 and SLC3A2) is associated with poor prognosis and short BCSS, particularly in the highly proliferative BC subtypes.^9^

Besides metabolic reprogramming, immune evasion is also considered as an emerging hallmark of cancer.^1^ The role of immune cells in tumour evasion is increasingly barbed, as many tumours not only escape recognition by the adaptive immune response but also sometimes cooperate with the pro-tumorigenic immune cells to become invasive and more aggressive. Furthermore, there is a link between the two mentioned hallmarks, as changes in the tumour cell metabolism can influence the component and function of the inflammatory infiltrates.^21,32^ This study showed that altered glutamine metabolism, which was detected by the overexpression of the key glutamine transporters (SLC1A5, SLC7A5 and SLC3A2) was significantly associated with the existence of specific subtypes of immune cells, namely CD68 + macrophages, FOXP3 + regulatory T cells (Tregs), CD20 + B lymphocytes and PD1 + lymphocytes along with its tumour-expressing ligand (PDL1). However, no association was observed between the SLCs and CD3 + or CD8 + T lymphocytes.

Our previous study on the same BC cohort showed that the main component of the inflammatory infiltrates is the pan-T-lymphocyte population (CD3 + cells) along with CD8 + cells being more frequent than FOXP3 + cells. The CD68 + macrophages were more frequent while CD20 + B lymphocytes were the least.^33^ In this study, however, we observed that changes in the metabolic activity of the cancer cells, which is reflected by an increase in glutamine transport, derive specific components of immune cells, which were restricted to CD68 +, FOXP3 +  and CD20 + along with PD1 + cells. This indicates that in these circumstances, the antigen- presenting cells (APC), CD68 + cells, are recognised only by specific subpopulations of T- and B lymphocytes.

When different BC subtypes were examined, a significant association was observed in ER + highly proliferative/luminal B, TN and HER2 + tumours, but not the ER + low proliferative/luminal A subtype. Both luminal B and TN tumours showed associations with CD68 + macrophages. These two subtypes are aggressive, highly proliferative and exhibit high metabolic activity. Consequently, aggressive cancer cells secrete high levels of reactive oxygen species (ROS) in their microenvironment.^34^ The latter can cause a state of pseudohypoxia in the adjacent stromal compartment with concomitant upregulation of hypoxia-inducible factor 1α (HIF1α), known to induce the pro-tumorigenic CD68 + macrophages.^35,36^ The same scenario can be applied when amino acids, particularly leucine, activate mTORC1 that upregulates HIF1α.^37^ Previous studies have shown that PD1/PDL1 are mainly expressed in HER2 + and TN subtypes.^20,38,39^ This study further shows that the expression of PD1 and/or PDL1 is mainly associated with high SLC expression but restricted to HER2 + and TN tumours.

In this study, we observed high expression of the solute carriers in the stromal lymphocytes. This is expected as glutamine transporters are not only necessary for cancer cells, but they are also important for optimal lymphocyte proliferation and differentiation.^40–43^ In addition, macrophages may require glutamine as it is the main precursor for arginine.^44^ The latter can be catalysed by Arginase 1 to support cell proliferation and tissue remodelling.^45^

Indeed, the upregulation of glutamine transporters in the cancer cells and their neighbouring immune cells, might indicate that both cell types are substantially comparable in their requirements of amino acids, which can be obtained from the TME, to support their survival and proliferation. Furthermore, TME might be a source of stromal glutamine, as it has been found that the metabolic stress in TME triggers genomic instability, which subsequently acquires the non-malignant cancer-associated fibroblasts (CAF) a catabolic phenotype with enhanced macroautophagy. This catabolic state produces a nutrient-rich environment, with increased amounts of pyruvate, lactate, ketone bodies and glutamine.^46^ This phenomenon also substantiates the cancer-stromal symbiosis that subsequently supports cancer growth and progression.

We and others revealed that high expression of glutamine solute carriers is associated with poor patient outcome.^7,8,47^ Similarly, the presence of FOXP3 + and CD68 + cells also correlates with shorter survival.^33^ However, CD20 + cells tend to be associated with better survival.^33^ In this study, we showed that the co-occurrence of SLCs with FOXP3 + and CD68 + cells can predict shorter survival compared with the presence of one without the other, whereas the combination of the SLCs with CD20 + cells derives better patient outcome.

The association between PD1/PDL1 and survival in BC is controversial.^20,48–50^ This study, however, showed that the co-expression of SLCs with PD1 or PDL1 was associated with shorter distant metastasis-free survival and breast cancer-specific survival, indicating that patients with BC show an increase in their amino acid metabolic activity that might influence poor outcome in PD1/PDL1 + tumours.

We observed that targeting SLC7A5 by transient siRNA significantly reduced the expression of PDL1 in TN cells. This can be attributed to the role played by this protein in activating the mTORC1 pathway, through importing essential amino acids, such as leucine. This might take place in parallel with the activation of mTORC1 through the AKT–mTOR signalling pathway, which is previously identified as a tight regulator of PDL1 expression in several cancers, including TNBC.^38,51,52^

Although clinical trials with monoclonal antibodies targeting PD1/PDL1 interaction have shown promising results, with durable responses, in several human cancers,^53–55^ not all patients respond to this targeted therapy. Therefore, it is critical to find effective approaches that could allow personalisation of treatment of PD1/PDL1 + tumours. This study not only provides clinical evidence that SLCs in BC could aid the personalisation of anti-PD1/PDL1 inhibition therapies, but it also emphasises that targeting the amino acid transporter, SLC7A5, along with the anti-PDL1 immunotherapy could be considered as a novel approach to synergistically enhance the therapeutic effect.

## Conclusion

This study revealed that there are associations between the two cancer hallmarks, metabolic reprogramming and immune evasion. Altered glutamine pathways in cancer cells can derive specific subtypes of inflammatory infiltrates, which act either with or against the aggressiveness and progression of the BC cells. Targeting both SLC7A5 and PD1/PDL1 can be a new approach that will counteract the highly proliferative and aggressive BC subtypes.

## Supplementary information

Supplementary material

## Data Availability

The data sets generated during this study are available from the corresponding author on reasonable request.
