# Supplementary material for: Enhanced glutamine uptake influences composition of immune cell infiltrates in breast cancer
Source: Br J Cancer. 2019 Dec 10;122(1):94–101. doi: 10.1038/s41416-019-0626-z (PMC6964696; doi:10.1038/s41416-019-0626-z)
Supplement: Supplementary file 1 — Supplementary material [file 41416_2019_626_MOESM1_ESM.pdf]

Supplementary table 1: Clinicopathological parameters of the Nottingham BC series

Parameters	Nottingham TMA series n (%)
**Tumour size**	
≤ 2cm	658 (51.6)
> 2cm	616 (48.4)
**Grade**	
1	207 (16.3)
2	415 (32.7)
3	649 (51.1)
**Tumour type**	
Ductal (including mixed)	1052 (82.6)
Lobular	115 (9.0)
Medullary-like	34 (2.7)
Miscellaneous	9 (0.7)
Special type	64 (5.0)
**Lymph Node Stage**	
1	774 (60.9)
2	396 (31.2)
3	101 (7.9)
**Follow-up Status**	
Alive	650 (51.0)
Died from Breast Cancer	425 (33.4)
Died from other causes	199 (15.6)
**ER**	
Negative	325 (25.8)
Positive	934 (74.2)
**PgR**	
Negative	509 (41.8)
Positive	709 (58.2)
**HER2**	
Negative	1051 (86.7)

Positive	161 (13.3)

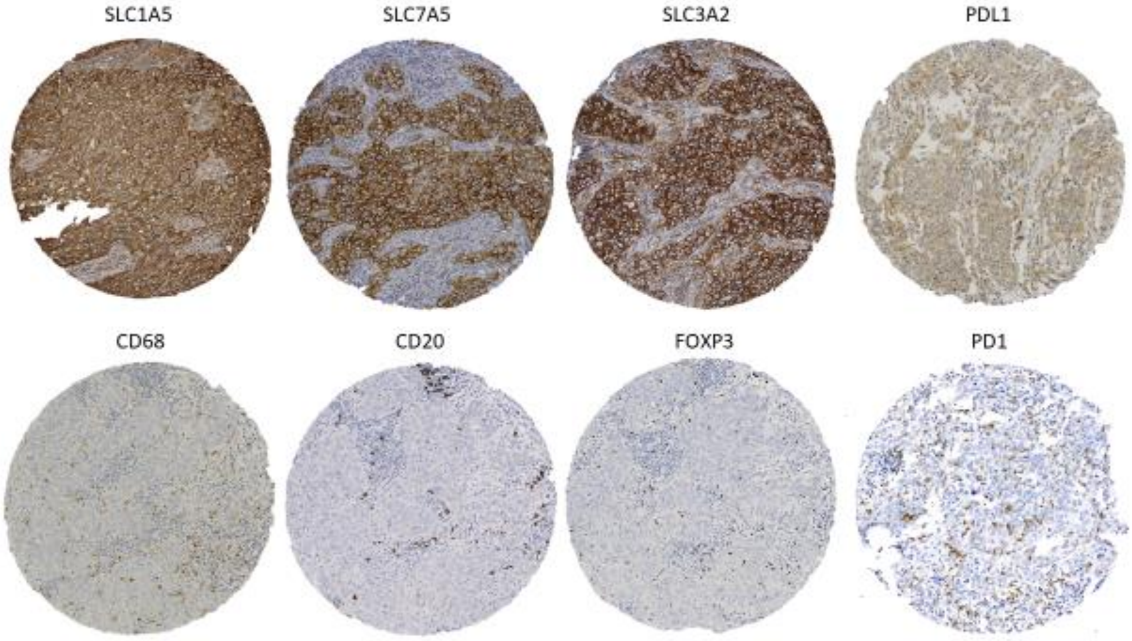

Supplementary figure 1. Immunohistochemical expression of SLC1A5, SLC7A5, SLC3A2 and PDL1 in invasive BC tumour cells (upper panel). Immunohistochemical expression of CD68, CD20, FOXP3 and PD1 in lymphocytic infiltrates of the invasive BC cores (lower panel).

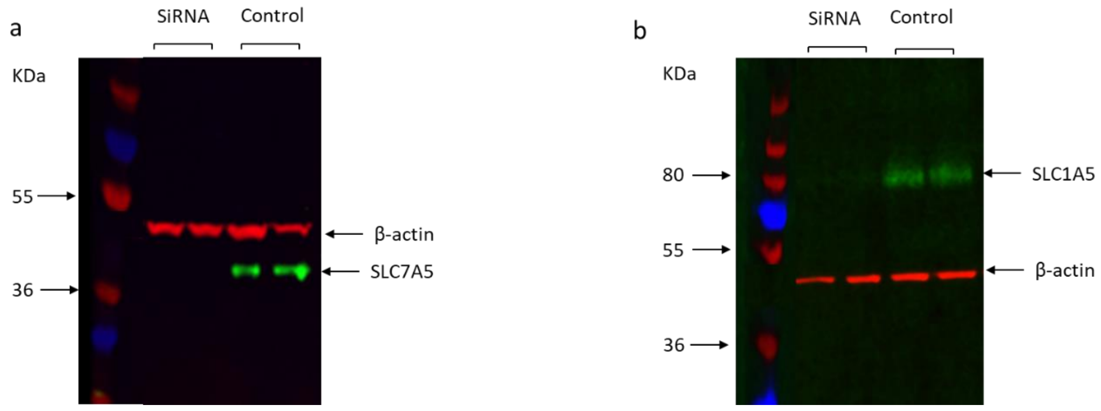

Supplementary figure 2. Knockdown of SLC7A5 and SLC1A5 in MDA-MB-231 cell lines. A) SLC7A5 expression in MDA-MB-231 transfected with SLC7A5 SiRNA and control (un-transfected) cells. B) SLC1A5 expression in MDA-MB-231 transfected with SLC1A5 SiRNA and control (un-transfected) cells.

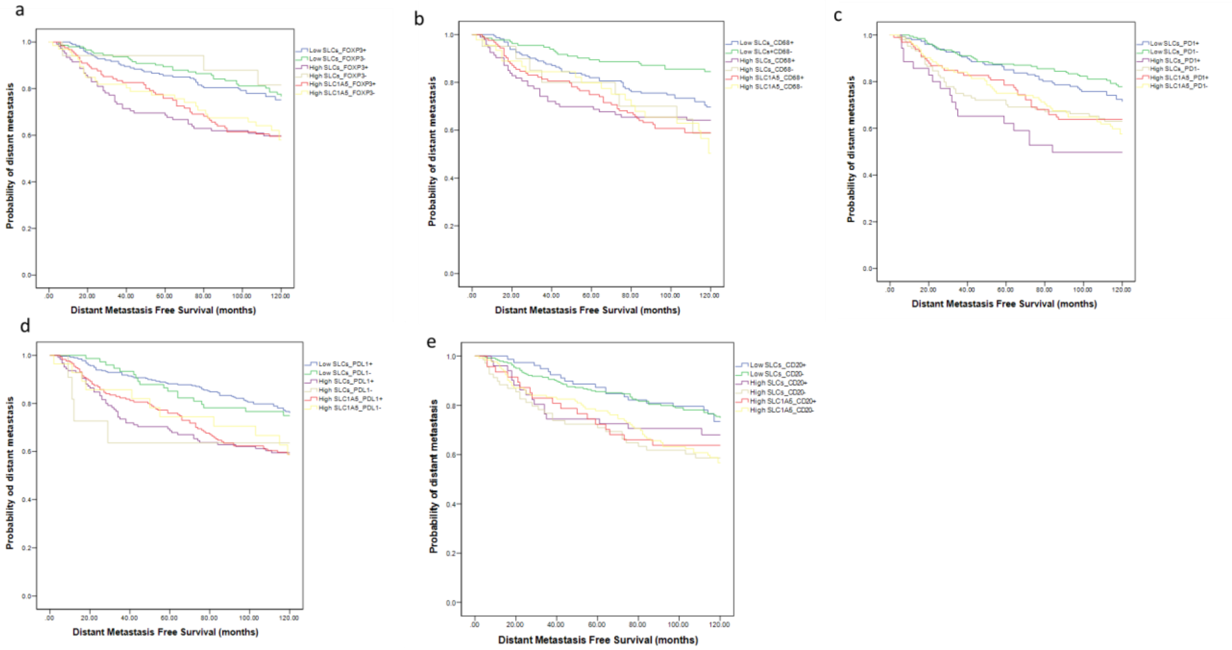

Supplementary figure 3. Distant metastasis Free Survival in SLCs and immune markers co-expression. A) SLCs-FOXP3. B) SLCs-CD68. C) SLCs-PD1. D) SLCs-PDL1. E) SLCs-CD20.
